# Teucrium polium: new toxic effects added to the literature: a case report

**DOI:** 10.1097/MS9.0000000000001760

**Published:** 2024-01-25

**Authors:** Ali Ghassa, Mousa Ismail, Leen Alabd Alrazak Alani

**Affiliations:** a Faculty of Medicine, Damascus University; b Department of Cardiology; c Internal Medicine Resident, Alassad University Hospital, Damascus University, Damascus, The Syrian Arab Republic

**Keywords:** cardiac toxicity, environmental toxicity, hepatotoxicity, herbal medicine, nephrotoxicity, teucrium polium

## Abstract

**Introduction and importance:**

Teucrium polium is one of the aromatic plants that grows in the Mediterranean region, and had been used as an herbal treatment for diabetes due to its hypoglycaemia effect. Although this plant is being studied now for its therapeutic role, its side effects are not taken enough into consideration, so this unique case can shed the light on serious toxic effects of this plant.

**Case presentation:**

A 68-year-old woman presented to the hospital with generalized fatigue, malaise, nausea, vomiting, abdominal pain, polydipsia, polyuria, breathlessness, and no defecation for 2 days after drinking big amounts of teucrium polium. The diagnosis was diabetic ketoacidosis (DKA), complete heart block, acute liver and kidney damage, and urinary tract infection (UTI). The patient was admitted to the ICU and treated for the DKA with an insulin pump, an antibiotic treatment for UTI, in addition to a dopamine pump and atropine, and then a temporary pacemaker was placed. The patient’s DKA, liver and kidney damage were improved on day 9, heart rate returned normal and she was discharged to insert a permanent pacemaker. However, the patient passed away at the end.

**Clinical discussion:**

Most studies made on this plant focused on the hypoglycaemia effect, with no attention to its toxic effects, so only few studies showed that teucrium polium can cause hepatic, renal toxicity and hyperglycaemia and most of them were studied in animals. While cardiac toxicity has never been noticed before.

**Conclusion:**

For this reason, herbal treatment should be used with caution to avoid catastrophic side effects.

## Introduction

Highlights

- Teucrium polium is a traditional herbal treatment for diabetes mellitus and many other conditions.
- The efficacy of teucrium polium in lowering blood glucose has been shown in many studies.
- Only few studies mentioned the harmful effects of teucrium polium, and those were mostly experienced in animals.
- This unique case revealed new toxic side effects of this plant on blood sugar, kidneys, liver and the heart.
- Any herbal treatment—if used inappropriately—will cause catastrophic side effects.

Diabetes mellitus is one of the most common diseases worldwide that is spreading exponentially and costing the health sector a lot. It is characterized by hyperglycaemia, decreased insulin levels, and hyperlipidemia, and has many serious negative effects on the body. One of the most important mechanisms that helps in developing diabetic complications and insulin resistance is oxidative stress, which occurs when the generation of free radicals exceeds the antioxidant defense system^1–3^. Hypoglycaemia oral drugs and insulin injections are used as treatment, however, these drugs have side effects, for instance, syncope, vertigo, anxiety, nervousness, depression, diarrhoea, nausea, and vomiting. This is why people try to find herbal medication with fewer adverse effects to treat diabetes, and one of these used herbs is teucrium polium. Several studies showed that it has a hypoglycaemia effect^3^. Although this plant is being studied now for its therapeutic role, its side effects are not taken enough into consideration, so in this paper, we present a rare case of a diabetic woman who stopped her diabetes treatment and decided to have large quantities of the mentioned plant. A week later, she came to the hospital with toxic manifestations; some of these are being mentioned for the first time in the literature.

## Case presentation

A 68-year-old woman presented to our emergency department with general malaise and fatigue. She was known to have type II diabetes mellitus controlled with insulin and metformin. In addition to that, she was on an oral beta blocker (metoprolol 50 mg a day) and self-administered a regular dose of aspirin (81 mg a day). The complaint started a week before admission when she discontinued the insulin and decided to begin with herbal treatment instead, which is teucrium polium. The patient used to soak the plant in hot water and drink three large cups daily for a week. Subsequently, she had general fatigue, nausea, vomiting, abdominal pain, polydipsia, polyuria, breathlessness, and no defecation for 2 days. The family confirmed that the patient did not have any heart conditions before.

On physical examination, vital signs were taken: (blood pressure: 190/50, heart rate: 34/min, O_2_ saturation: 97%, Respiratory rate: 19/min), besides dry mucus membranes and grade II pitting oedema that were noticed.

**Figure 1:**
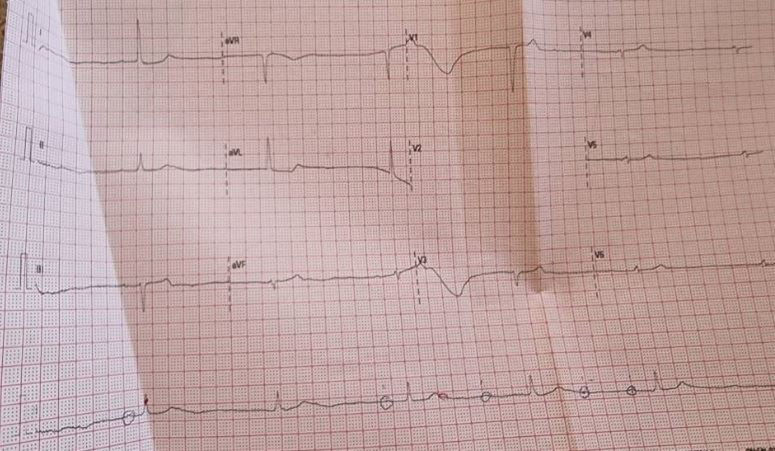
Electrocardiogram showing the initial status of the patient when first admitted to the hospital, it is described as complete heart block.

Investigations started with laboratory tests (shown in Tables 1–3). Diabetic ketoacidosis was clear, according to Glucose, ketone, and arterial blood gases. The increase in white blood cells besides the urinalysis guided us to urinary tract infection (UTI). The elevation of aspartate aminotransferase, alanine transaminase, creatinine, and urea was evidence of liver and kidney injury. Electrocardiogram (ECG) showed complete heart block (Fig. 1), while echocardiogram revealed severe cardiac hypokinesia with ejection fraction of 55%, Grade 2 mitral valve regurgitation, aortic valve regurgitation, and an inferior vena cava of 17 mm. The final diagnosis was diabetic ketoacidosis (DKA), complete heart block, acute liver and kidney damage, and UTI.

**Table 1:** Blood tests

Laboratory test	Test value	Normal values
WBC	14.5×10^3^/mm^3^	4–11×10^3^/mm^3^
Neutrophils/lymphocytes	77%/15%	40–70%/20–40%
Red blood cells	4.39×10^9^ /mm^3^	4–4.8×10^9^ /mm^3^
Haemoglobin	11.9 g/dl	12–14 g/dl
HT	38.8%	37–42%
MCV	88.4 fl	82–96 fl
MCH	27.1 pg	27.5–33.2 pg
PLT	240.000/mm^3^	150–450/mm^3^
Glucose	721 mg/dl	75–110 mg/dl
ALT	430 IU/l	10–25 IU/l
AST	239 IU/l	10–36 IU/l
Urea	78 mg/dl	10–50 mg/dl
Creatinine	1.86 mg/dl	0.6–1.13 mg/dl
LDH	589 U/l	105–233 U/l
CK	219 U/l	30–145 U/l
Total protein	6.2 g/dl	6–8.3 g/dl
Albumin	3.1 g/dl	3.4–5.4 g/dl
Calcium	7.9 mg/dl	8.6–10.3 mg/dl
Phosphorus	3.9 mg/dl	2.8–4.5 mg/dl
Cholesterol	119 mg/dl	<200 mg/dl
HDL	40 mg/dl	35–80 mg/dl
LDL	75 mg/dl	Below 100 mg/dl
Uric acid	9.2 mg/dl	3.5–7.2 mg/dl
Na	123 mEq/l	135–145 mEq/l
K	5.6 mEq/l	3.5–5 mEq/l
Cl	92 mEq/l	95–105 mEq/l
Ketone	Positive	

ALT, alanine transaminase; AST: aspartate aminotransferase; CK, creatine kinase; HDL, high-density lipoprotein cholesterol; HT, hematocrit; LDH, lactate dehydrogenase; LDL, low-density lipoprotein cholesterol; MCH, mean corpuscular hemoglobin; MCV: mean corpuscular volume; PLT, platelets; WBC, white blood cell.

**Table 2:** Arterial blood gases

Test	Test value	Normal value
PH	7.251	7.35–7.45
PO2	70 mmHg	80–100
PCO2	24.5 mmHg	35–45
HCO3	10 mmol/l	22–26 mmol/l
Anion gap	21	8–12

**Table 3:** Urinalysis

PH	5
Specific gravity	1.025
RBC	125
WBC	300
Haemoglobin	+
Nitrate	+
Uric acid	++
Bacteria	++
Glucose	+++
ketone	++

RBC, red blood cell; WBC, white blood cell.

**Figure 2:**
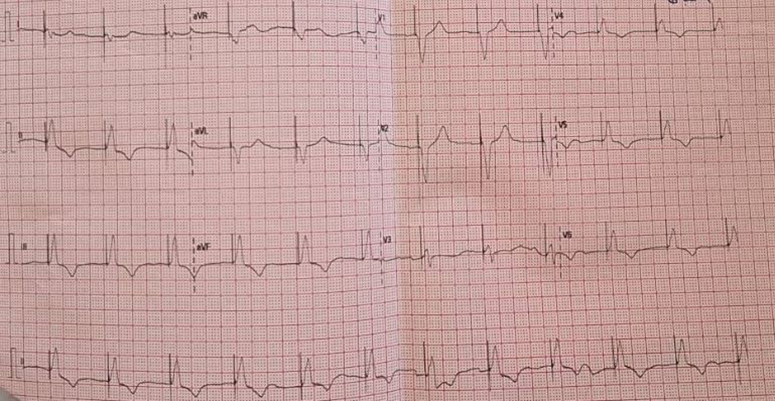
Electrocardiogram showing the rhythm after the temporary pacemaker, it is described as the following: regular rhythm 70/min, occasional p waves not related to QRS, inverted T waves in II, III, aVF, and V3 toV6, pacemaker on underlying complete block.

The patient was admitted to the ICU and treated for the DKA with an insulin pump, and an antibiotic treatment for UTI, in addition to a dopamine pump and atropine. After 24 h, the anion gap returned to normal, the blood pressure was 120/60, but her heart rate was still 34/min. On day 3, a temporary pacemaker was placed with no effect on the heart to be mentioned. On day 5, alanine transaminase and aspartate aminotransferase dropped to 117 and 46, respectively, but creatinine and urea rose to 2 and 90 in that order. Afterward, she had a 1-min sudden cardiac arrest and syncope that resulted in aspiration pneumonia, so metronidazole was added to the treatment, and an improvement in heart rate was noticed, which reached 73 beats per min (Fig. 2). On day 9, liver enzymes and kidney damage improved, and the lab values were as follows: aspartate aminotransferase: 62 IU/l, alanine transaminase: 17 IU/l, Cr: 1.0 mg/dl, Urea: 77 mg/dl, Glucose: 160. The patient was discharged to insert a permanent pacemaker. After that, we have heard from the family that she passed away, and we could not know any information about the cause of death.

## Discussion

Teucrium polium or germander is a type of peppermint, a kind of aromatic plant that mainly grows in the Mediterranean region^4,5^. Germander has been used as an herbal medicine throughout centuries for gastrointestinal disorders, kidney problems, wound healing, and diabetes, and mostly by Mediterranean people^4^. Throughout history, natural products helped in manufacturing anti-diabetic drugs, for example, the discovery of metformin from Galega Officinalis^5^.

Several studies proved that teucrium polium has many bioactive products such as terpenoids, flavonoids, iridoids, diterpenes, and phenylethanoids glycosides, these studies also proved that the plant has hypoglycaemia as well as increasing insulin effect^3,5^. The insulinotropic effect has been observed to be accomplished due to flavonoids and specifically apigenin. However, it has been noticed that giving high doses and concentrations above 1000 microgram/millilitre lowers the hypoglycaemia effect because of the cytotoxic influence^5^. Although the main effect is lowering blood glucose, only two cases of hyperglycemic effect have been mentioned in animal trials^5^.

Hepatotoxicity was mentioned for the first time in 1992 as several cases of acute liver injury occurred after taking teucrium polium for several years. The toxicity results from the reactivation of epoxide receptors that have been generated by the CYP3A metabolism of its constituents neoclordaneditrepenoids and these epoxides affect glutathione and cytoskeleton proteins of the hepatic cell which will end in apoptosis. Elevated liver enzymes will be seen as well as antinuclear antibodies (ANA), anti-smooth muscle antibodies, and anti-mitochondrial M2 antibodies. A specific antibody (anti-microsomal epoxide hydrolase), which is an antibody directed against epoxide hydrolase on the hepatic cell surface can be seen. Most liver injury cases resolve on its own, although in some cases the situation can develop to cause serious conditions that might need emergent liver transplantation^6^.

Teucrium polium has a toxic effect on the kidneys as a study conducted on rats proved that kidney damage is dose-related. The bigger the given amount is, the more probable the injury happens. Additionally, kidney damage increases after stopping the intake of the herb^7^, which occurred exactly in our case. A similar case of a woman with acute kidney failure has occurred after using big amount of teucrium polium^8^. However, nephrotoxicity due to this plant has only been mentioned few times in the literature^7,8^.

Teucrium polium has a positive chronotropic and ionotropic effect, in addition to the hypotensive effect because the plant extract is an angiotensin II receptor blockade and has a cardiac baroreflex effect during hypertension. Therefore, the extract can cause non-significant heart rate depression. In addition, in a study made by Seed Niazmand and colleagues showed that teucrium polium has no effect on the heart rate^9–11^. However, in our case this caused severe bradycardia.

To our knowledge, hyperglycaemia and cardiac toxicity due to teucrium polium have never been reported in humans before, making this case unique.

## Conclusion

Although herbal medicine has many benefits, it can be misused, causing several side effects. Therefore, herbal drugs should be utilized according to medical consult to avoid serious illnesses.

## Ethical approval

Not required for case reports at our hospital. Single case reports are exempt from ethical approval in our institution.

## Consent

Written informed consent was obtained from the patient to publish this report in accordance with the journal’s patient consent policy.

## Source of funding

The authors received no funding regarding the publication of this article.

## Author contribution

A.G. gathered the data, researched the literature, and wrote the manuscript. M.I. and L.A.A.A. diagnosed and treated the patient and reviewed the article for scientific adequacy. All authors reviewed and approved the final manuscript before submission.

## Conflicts of interest disclosure

The authors declare that there is no conflict of interest to be reported.

## Research registration unique identifying number (UIN)

Not required for this case report.

## Guarantor

Mousa Ismail.

## Data availability statement

All data generated during this study can be accessed through direct communication with the corresponding author and the agreement of all research team members.

## Provenance and peer review

Not commissioned, externally peer-reviewed.
